# The challenges of eating out for young people with eating disorders: a thematic analysis of the perspectives of young people, parents and carers, and clinicians

**DOI:** 10.1186/s40337-025-01471-z

**Published:** 2025-12-16

**Authors:** Lydia Shackshaft, Laura Chapman, Annabelle Hook, Lucy Biddle, Lucy Yardley, Tamsin Ford, Angela Attwood, Ian Penton-Voak, Mel Slater, Emily Rothwell, Stella Reeves, Alys Grant, James Downs, Gillian Combe, Sam Clark-Stone, Trinisha Govender, Anne Stewart, Paul Moran, Helen Bould

**Affiliations:** 1 Centre for Academic Mental Health, Population Health Sciences, Bristol Medical School, University of Bristol, Bristol, UK; 2 University Hospitals Bristol and Weston NHS Foundation Trust, Bristol, UK; 3 Oxford Health NHS Foundation Trust, Oxford, UK; 4 NIHR Biomedical Research Centre, University Hospitals Bristol and Weston NHS Foundation Trust and University of Bristol, Bristol, UK; 5 School of Psychological Science, University of Bristol, Bristol, UK; 6 School of Psychology, University of Southampton, Southampton, UK; 7 Department of Psychiatry, University of Cambridge, Cambridge, England UK; 8 Virtual Bodyworks S.L., Barcelona, Spain; 9 Department of Clinical Psychology and Psychobiology, Universitat de Barcelona, Barcelona, Spain; 10 Greater Manchester Mental Health Foundation Trust, Manchester, UK; 11 Expert By Experience, Bangor, Wales UK; 12 Expert By Experience, Canterbury, UK; 13 Expert By Experience, Cardiff, Wales UK; 14 Gloucestershire Health and Care NHS Foundation Trust, Gloucestershire, UK; 15 Department of Psychiatry, University of Oxford, Oxford, UK

**Keywords:** Eating disorders, Adolescents, Young adults, Lived experience, Qualitative research, Thematic analysis, Social eating

## Abstract

**Background:**

Social eating is a key aspect of recovery for many individuals with an Eating Disorder (ED). To develop effective interventions to support recovery of social eating we need to understand the challenges that people with ED face when eating in public spaces. This study was conducted in the context of the development of a virtual reality graded-exposure café intervention for people with ED. The current analysis explores stakeholder perspectives on the challenges that people with ED face in café environments.

**Methods:**

People with lived experience of ED (n = 15), parents/carers (n = 4) and clinicians (n = 6) took part in semi-structured focus groups and 1:1 interviews. Transcripts were analysed thematically.

**Results:**

We identified six major themes: (1) Facing the unexpected and unknown; (2) Cafés elicit difficult emotions; (3) Challenges are highly individual; (4) Challenges relating to the physical café environment; (5) Challenges of social interactions in cafés; (6) Challenges of the process of choosing and consuming food and drink.

**Conclusions:**

This study highlights the challenges cafés present for people with ED, many of which also apply to other social eating scenarios. These findings will enable more targeted support and development of novel interventions to help people with ED return to social eating.

**Supplementary Information:**

The online version contains supplementary material available at 10.1186/s40337-025-01471-z.

## Abstract (plain language summary)

Many people with an Eating Disorder (ED) find it difficult to eat in social settings such as cafés, and to eat socially with family and friends. For many individuals with an ED being able to do this is an important part of recovery. To help us provide targeted support in this area we first need to understand the challenges that people with ED face when eating socially in public spaces.

We conducted interviews and focus groups with people with lived experience of ED (aged 14–25 years), parents/carers, and clinicians. Participants described cafés as difficult places where people with ED had to face unexpected challenges, and may experience intense difficult emotions including feeling anxious, stressed and overwhelmed. The noise and busyness of the physical café environment contributed to these feelings. Interactions with other people in cafés also posed a challenge including in relation to fears about judgement of appearance and food choices. Challenges around looking at menus with and without calories, and choosing and ordering food were also highlighted, as well as actually consuming food and drink. Despite common themes, challenges faced are individual and may depend on ED diagnosis and stage of recovery.

Although this study focused on the challenges faced in cafés to inform the development of a virtual reality (VR) café intervention, many of the findings relate to any social eating or public scenario. This detailed understanding about the challenges people with ED face in these scenarios will enable clinicians and potentially individuals’ wider support networks to provide more targeted support of social recovery, as well as informing the design of our virtual reality café.

**Supplementary Information:**

The online version contains supplementary material available at 10.1186/s40337-025-01471-z.

## Background

Eating Disorders (ED) are serious mental illnesses, with a mortality rate over three times higher than the general population [1]. Despite increasing recognition of the impact of ED, global incidence rates are rising [2, 3]. ED affect all ages and genders, most commonly adolescents and young adults [4, 5]. Approximately a third of individuals do not fully recover: the worst outcomes are seen in Anorexia Nervosa (AN) with a median illness duration of ten years [5]. Many relapse within two years of treatment [2]. Development of novel treatments is therefore a priority to prevent adverse effects on young people’s physical, emotional, social, and educational development.

Impaired social and emotional functioning are frequently seen alongside ED, and are both risk factors for, and consequences of AN [6]. A significant aspect of this socio-emotional phenotype is the high comorbidity of ED with anxiety disorders, particularly Social Anxiety Disorder (SAD) and Obsessive–Compulsive Disorder (OCD), which commonly predate future ED diagnoses [7–9]. Social anxiety symptoms may act as both risk and maintenance factors for ED, with evidence to suggest that social fears related to eating and drinking in public may act as an illness pathway from SAD to ED and vice versa [7, 10]. Similarly, the shared symptom of intrusive thoughts appears to contribute to the maintenance of comorbid OCD and ED, which includes concerns about being seen eating in public [11, 12]. It has been suggested therefore that concerns about social eating may be a transdiagnostic symptom central to the comorbidity ED with multiple anxiety disorders [12]. Regarding social functioning, whilst relationship loss and social isolation are core maintaining factors for AN, social support and inclusion are important for recovery [5, 13, 14]. This pattern of social isolation is seen across the spectrum of ED, with low subjective social support reported by individuals with AN, Bulimia Nervosa (BN), and Binge-Eating Disorder (BED) [15]. Despite the paucity of research on other ED diagnoses [2], OSFED (Other Specified Feeding or Eating Disorder) appears to be associated with similar functional impairment and psychological symptoms as ‘classical’ ED diagnoses [16], and social withdrawal has been noted as a particularly significant psychosocial issue for people with ARFID [17]. Sharing meals with family and friends is an important activity across cultures, and has relational benefits for forming and reinforcing relationships, building social networks for socio-emotional support, and increasing life satisfaction [18]. Difficulties with social eating, including sharing meals with family and friends, as well as eating out with others, therefore contribute significantly to the social impairment experienced by people with ED [19]. For many, regaining the ability to comfortably eat with others in social settings is an important part of recovery [20], as is re-building supportive relationships and a sense of belonging [13, 21]. However, there is minimal research regarding the challenges that social eating poses for people with ED, or how to support this behaviour [22].

For young people, social eating can occur in many settings beyond their home, including at school, friend’s houses, or out with friends at cafés and cinemas [23]. Young people describe eating out with friends as a focal point for shared experiences, a valued enjoyable activity, and opportunity for social connectedness [23]. Given its importance to people with lived experience (PWLE) [20], supporting them to regain an ability to eat out, and therefore enable them to engage in this key aspect of social eating, is an important area for further study. To develop effective interventions we first need to understand the challenges that people face when eating in public spaces.

## Aims of the current study

This study was conducted in the context of a broader project developing novel virtual reality (VR) interventions for people with ED. Regarding treatment of mental health conditions, VR offers an avenue for people to develop mastery of simulated difficult situations that can subsequently be transferred to real life [24]. Previous qualitative work with PWLE and clinicians exploring the potential use of VR as a treatment adjunct for ED has highlighted that it could be beneficial to practice scenarios that involve eating out, specifically in cafés [25].

Participants in the present study were therefore recruited as part of a qualitative study to help inform the development of a VR graded-exposure café scenario using the person-based approach [26, 27]. PWLE, parents and carers of individuals with an ED, and clinicians with relevant experience took part in focus groups or 1:1 interviews exploring perspectives on the use of a VR café as an intervention for ED; the challenges they felt would be beneficial to practice in a VR café, and integration with existing treatments. The richness of the data collected was such that we conducted the current analysis to specifically explore stakeholder perspectives on the challenges that people with ED face in a café environment.

## Methods

### Participant recruitment and selection

We included PWLE aged 14–25 years with any current or previous ED, who found going to, or eating/drinking in, cafés challenging. Parents/carers were included who reported current or previous experience caring for a person with an ED who found going to or eating/drinking in cafés challenging. Clinicians were included who were currently working as a health professional and had experience treating people with ED. We excluded individuals undergoing inpatient treatment in a general or psychiatric hospital, individuals not fluent in English, those who did not have access to an internet-enabled device and private space to join a focus group or interview, and members of the project’s Patient and Public Involvement and clinician advisory groups.

PWLE and parents/carers were recruited via social media, advertisement by UK ED charities, poster advertisement in public places, and snowballing. Clinicians were recruited via social media and snowballing through professional contacts. Participant screening to determine eligibility was completed via online SurveyMonkey questionnaires. Purposive sampling aimed to ensure diverse representation of age, gender, ethnicity, ED type, and clinician professional background. Selected participants were invited to attend a video call and provide photographic ID to confirm their identity, during which capacity/competence to provide informed consent was assessed.

We aimed to recruit approximately ten PWLE, four parents/carers and four clinicians in line with previous intervention development work using the person-based approach to capture all relevant perspectives [26–28]. Pragmatic considerations also influenced sample size, namely that recruitment continued until participants reflected sufficient diversity of characteristics. This was defined as inclusion of PWLE across key demographic categories where different experiences might be expected, and inclusion of those often under-represented in ED research. Specifically, we sought to include participants across the whole eligible age range (early adolescence 14–15 years, late adolescence 16–19 years, and emerging/young adults 20–25 years), of both female and male gender, with representation from minoritised ethnic backgrounds, and with experiences across the diagnostic spectrum of ED including AN, BN, BED, ARFID and OSFED. After early interviews and focus groups we reviewed the demographic representation and sought additional participants from under-represented groups.

### Participant characteristics

In total, 25 individuals participated in a focus group or 1:1 interview (15 PWLE, four parents/carers, and six clinicians). The majority of participants were female (PWLE: n = 12; Parents/carers: n = 3; Clinicians: n = 5) and White/ White British (PWLE: n = 12; Parents/carers: n = 4; Clinicians: n = 6). Participants had collectively experienced or supported someone with a range of ED diagnoses including Anorexia Nervosa (AN), Bulimia Nervosa (BN), Binge-Eating Disorder (BED), Avoidant/Restrictive Food Intake Disorder (ARFID) and Other Specified Feeding or Eating Disorder (OSFED), with multiple participants describing experiences from across the spectrum of eating behaviours. Clinicians were from a range of medical, nursing, clinical psychology, and allied health professional backgrounds. Personal lived experience of ED was also disclosed, unprompted, by a small number of parent/carer and clinician participants. Given the narrow aim of the study, the relatively dense specificity of the sample, and an overall strong quality of dialogue between researchers and participants, we considered the recruited sample to hold sufficient information power [26, 29]. Participant characteristics are shown in Table 1.

**Table 1:** Focus group and interview participant characteristics

Characteristics	Values
**People with Lived Experience of Eating Disorders (n = 15)**	
**Age**	
Age, range (years),	14–25 (x̄ = 20)
**Ethnicity**	
White/White British, n	12
Black British/Asian/mixed/other, n	3
**Gender**	
Female, n	12
Male, n	2
Non-binary, n	1
**Eating disorder diagnosis or behaviours (current or previous)**	
Anorexia Nervosa, n	12
Bulimia Nervosa/Bulimic behaviours, n	4
Binge eating disorder/binge eating behaviours, n	2
Avoidant/restrictive food intake disorder, n	3
Orthorexia, n	2
Other specified feeding or eating disorder, n	2
Duration of eating disorders	
Recovered from eating disorders (self-report), n	7
Duration of eating disorders, range (years)	1–14 (x̄≈5)
**Parents/carers (n = 4)**	
**Age**	
Age, range (years)	49–63 (x̄ = 56)
**Ethnicity**	
White/White British, n	4
**Characteristics of young person cared for**	
Gender	
Female, n	3
Male, n	1
Age, range (years)	16–22 (x̄ = 19)
Ethnicity	
White/White British, n	4
Eating disorder diagnosis or behaviours (current or previous)	
Anorexia Nervosa, n	3
Bulimia Nervosa, n	1
**Clinicians (n = 6)**	
**Age**	
Age, range (years)	26–65
**Ethnicity**	
White/White British, n	6
**Gender**	
Female, n	5
Gendervague, n	
**Duration of experience working with people with eating disorders**	
Duration, range (years)	1–23 (x̄≈6)

x̄ = mean average≈ = estimated value

### Focus group and interview procedure

Participants attended a focus group or 1:1 interview (via videoconferencing software) based on personal preference and availability. Of the 25 participants, eight PWLE participated across three focus groups (n = 3; 3; 2), whilst 17 participants took part in 1:1 interviews. These were conducted by LC, LS or HB (focus groups had two facilitators), lasted an average of 41 min (range 24–65 min) and were semi-structured, based on a topic guide (see Additional file 1). Audio was recorded using an encrypted digital recording device. A debrief email was sent following participation with a £20 e-voucher and signposting to relevant support services.

### Analysis

Interview and focus group recordings were transcribed verbatim, anonymised and imported into qualitative data analysis software NVivo 1.7.1 [30]. Thematic analysis (TA) was conducted following the six stages outlined by Braun and Clarke [31]. A critical realist approach was adopted, guided by the conceptualisation of experiential and inferential themes described by Wiltshire and Ronkainen [32].

Following data familiarisation, LS inductively coded all transcripts. A portion of transcripts (n = 3) were independently coded by LC, recognising the influence of differing personal and professional experiences. This enabled discussion of differing interpretations to reach consensus and develop a rich understanding of the data [32]. Codes were used to develop initial experiential themes, closely describing participants’ perspectives. An iterative process of reviewing and refining themes and subthemes through discussion between LS, LC and HB provided a comprehensive understanding of the dataset. Through this process, abductive reasoning led to the development of more abstract and conceptual inferential themes [32].

### Positionality and reflexivity

Researchers reflected on their experiences and assumptions throughout the TA process, recognising that researcher subjectivity influences all stages, but can be a valuable source of knowledge to interpret participant’s accounts. LS is a resident doctor with experience caring for patients with ED in acute hospital settings, and has lived experience of an ED. LC is an early career researcher with a background in psychology and a research focus on ED, who also has lived experience of an ED. HB is an associate professor in child and adolescent psychiatry, with both clinical and research expertise in ED and child and adolescent mental health. Independent coding of a portion of transcripts by LC, and regular meetings between LS, LC and HB (all of whom were familiar with the dataset) to discuss the evolving analysis enabled us to use these various experiences to share understandings and interpretations of the data.

### Ethical approval

Ethical approval was granted by the University of Bristol Faculty of Health Sciences Research Ethics Committee (reference: 12426). Participants completed a consent form prior to participation, and parental assent was additionally required for participants aged 14–15 years. To ensure the safety and wellbeing of participants, a distress protocol was designed. This included focus groups being co-facilitated by a mental health clinician, enabling additional support to be offered if needed. HB (consultant psychiatrist) was available during 1:1 interviews via telephone call in the event of any concerns about a participant’s wellbeing.

## Results

### Major themes from the thematic analysis

**Fig. 1:**
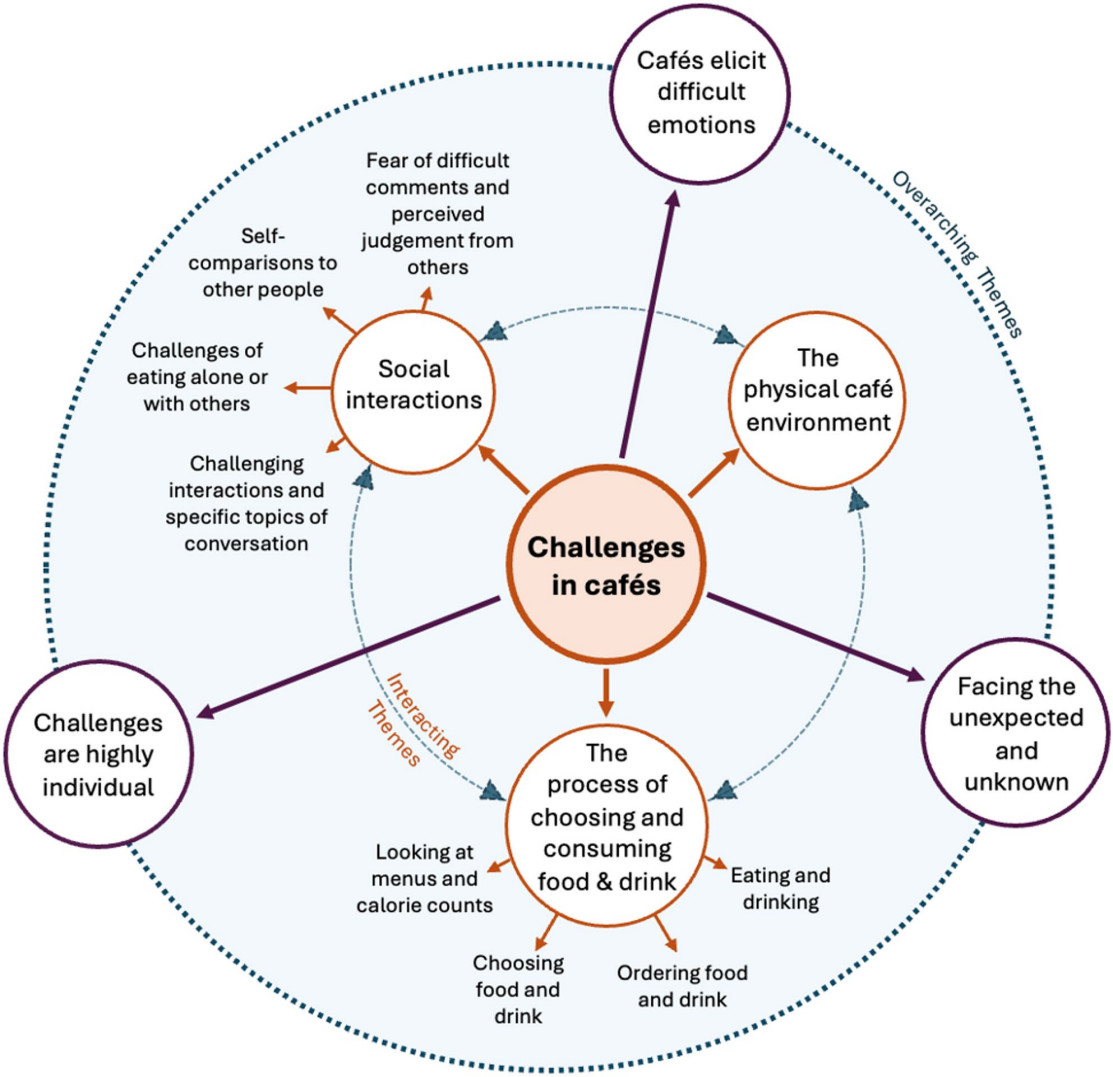
Thematic map demonstrating the six major themes identified, associated subthemes, and the relationships between them. Three experiential themes (orange) of the challenges associated with the café environment, social interactions, and the process of choosing and consuming food and drink were felt to be highly interlinked, with each aspect enhancing the challenges of the others. Facing the unexpected and unknown, cafés eliciting difficult emotions and challenges being highly individual formed discrete but overarching inferential themes (purple) that relate to the three more concrete challenges within the inner circle

We identified six major themes. Three themes were overarching and inferential in nature: (1) Facing the unexpected and unknown; (2) Cafés elicit difficult emotions; (3) Challenges are highly individual. Three were experiential in nature, relating to the challenges experienced in cafés, namely: (4) Challenges relating to the physical café environment; (5) Challenges of social interactions in cafés; (6) Challenges of the process of choosing and consuming food and drink. These themes, associated subthemes, and their interactions are demonstrated in a thematic map (Fig. 1).

### Facing the unexpected and unknown

Many of the challenges described were linked by an overarching theme (see Fig. 1) of cafés being places where participants faced the unexpected and unknown: *“…after saying all of this the big word that comes to me is uncertainty and unpredictability. I think those words relate to everything I’ve just said in the last ten minutes.”* (Cl-2)^1^^1^.

Facing the unexpected was an underlying difficulty unifying many of the challenges described relating to the physical café environment (see 3.5), social interactions (see 3.6), and choosing and consuming food (see 3.7): *“you have got the unexpected food, you have got lots of people, lots of noise, people could talk to you, people could not, you don’t know where you’re going to sit like there’s so many sort of variables I think, that’s what makes it quite daunting”* (PWLE-8). The fear of unknown food was referred to in many contexts (see 3.7): not being able to look at a menu in advance, unknown quantities and ingredients, unknown calories, unfamiliar foods not matching a meal plan, and food arriving that is different to expected: *“…that would be the end of the world like if they had put cream on it [iced coffee] and I hadn’t asked for that, that’s something I hadn’t expected and pre-empted and prepared for…”* (PWLE-9)^2^^2^.

PWLE described going to a new café as more difficult than returning to somewhere previously visited, and that this was likely the result of the sense of safety from routine. Chain cafés were largely felt to be easier with more predictability, familiar environments and similar menus: *“…it’s probably easier with a chain café because independent cafés are all very different to each other just in terms of how they look and how they feel, whereas chain cafés tend to have a bit more cohesiveness to them.”* (PWLE-15). One participant with BED reported that new cafés were easier due to the anonymity they experienced, and reduced fear of recognition and judgement from others (see 3.6.1).

### Cafés elicit difficult emotions

The experience of intense unpleasant emotions, most frequently feeling stressed and *“getting more and more overwhelmed, more and more anxious”* (PC-2)^3^^3^, formed a second overarching theme (see Fig. 1). These emotions resulted from combined environmental, social and food-related challenges (see 3.5, 3.6, and 3.7). Difficulties with noise, sensory overload and other people in the café (see 3.5) were frequently felt to be major contributors: *“…it’s your brain talking at you, and then the external stimuli, and then all the chattering going on, or someone trying to encourage you, it’s all those things, all together.”* (PWLE-4); as were looking at menus with calories and making decisions about food (see 3.7.1 and 3.7.2): *“She’d be looking at the calories and getting really upset about the calories, and then it would turn into fear and she would have a panic attack that there’s too many calories…”* (PC-3).

Feeling overwhelmed and anxious were described not only as a *consequence* of the other challenges in cafés, but also as having a *subsequent impact* on someone’s ability to deal with those challenges: one clinician spoke of how an *“over-stimulating”* sensory environment could *“raise anxiety and limit someone’s capacity to do something that’s really challenging.”* (Cl-2).

### Challenges are highly individual

Many participants highlighted that the challenges encountered depended on an individual’s ED diagnosis and behaviours, particularly when comparing AN and restrictive EDs to binge-eating and purging behaviours:

"…the expectations to even just eat in a normal amount of time...someone with anorexia, in particular, they might take an hour to eat that and potentially sometimes longer. Someone with a binge-eating disorder might feel terrified because they know how fast they eat and once they start eating, they will just keep going and going. For someone with bulimia, it will be that they want to obviously go and purge and they might have an awareness that people have been watching them." (Cl-5)

Two participants suggested that those with binge-eating behaviours might find cafés less challenging than those with restrictive ED, as these were thought to more commonly occur in private settings: *“that’s something that you can still work quite well in public …that’s such a private..that’s something that doesn’t happen in the public.”* (PWLE-2).

Challenges were also thought to change based on the stage of recovery: *“…it depends what stage in recovery you are in so, like some people just going into a café would be like, ‘whatever, that’s stupid, why am I practicing that?’ And it would be the ordering and eating, but for some people, at that moment in time, just going in would be hard.”* (PWLE-10).

Despite common themes, PWLE nevertheless emphasised that *“different people are going to have different struggles, different fears, different eating disorders”* (PWLE-12).

### Challenges relating to the physical café environment

Even entering a café environment was noted as a significant challenge: *“…I found even just being in a café environment really hard, like I wouldn’t even consider getting any of the food…”* (PWLE-10), and *“…she would be too scared to even go in sometimes”* (PC-3).

Participants highlighted the multitude of sensory stimuli of all modalities in café environments, including lighting, music, colours and smells, which contributed to the challenging emotions described in 3.3: *“…the sound of people chewing, multiple different conversations going, if there’s a coffee machine going or people coming in and out of the door and the door banging… the variety of smells can be really overwhelming.”* (Cl-5). Such sensory challenges heightened the difficulty PWLE experienced when tackling tasks in cafés such as decision making and eating (see 3.7): *“…if there was lots of noise going on then I’d find it harder to eat because I’d feel like I couldn’t focus on my food…”* (PWLE-15). All stakeholder groups noted the prevalence of neurodiversity amongst those with ED and its potential contribution to this sensory overload.

A busy environment with lots of people was commonly described by all stakeholder groups as another challenge of cafés. Some felt *“the hustle and the bustle”* (PC-3) in itself was challenging and stressful. For many, the difficulties of a busy café were associated with heightened social challenges (see 3.6); *“…when it is too crowded, they might think that there are too many more people to judge”* (Cl-1), and *“…being really busy means I have even more comparison…”* (PWLE-2). However, many also described difficulties of quiet cafés where they felt rushed to make decisions, or *“…feel on the spot and like can everyone see what they are eating and how they are eating.”* (Cl-1).

Participants described many additional challenging aspects of a café environment relating to contents and layout (Table 2).

**Table 2:** Example codes and participant quotes relating to the challenges of the contents and layout of café environments

Code describing a challenging aspect of a café environment	Example quote from participant
Seeing or being around food as a challenge	*“…just being around people eating and being around like cake and stuff, I know a lot of people struggle with that… I think just being around food can be really difficult.”* (PWLE-8)
Seeing food being prepared as a challenge	*“…being able to actually see, probably, especially the food… maybe how it’s being prepared, stuff like that are stuff that I personally would focus on a lot if I was in a café anyway.”* (PWLE-2)
Not being able to watch your order being prepared as a challenge	*“I like to watch what they’re doing, like, that they’re using the right things. So I think that could be a challenge, as well, to like not watch, or like, trust that they’re getting your order right…”* (PWLE-3)
Seeing your own reflection as a challenge	*“…I think it would be good if there’s like a cake counter to not have that reflective so that you don’t kind of see your own reflection in it.”* (PWLE-15)
Window seats as a challenge	*“some restaurants have windows next to window seats. This might also be challenging because not only can people in the café or restaurant see you but also people from outside can see you. You might feel also stressed about this.”* (Cl-1)
Seeing other people eat as a challenge	*“Having the smells, the sights and the sounds of other food like hearing somebody else eat or watching somebody else eat can be very difficult even if you’re not eating yourself.”* (Cl-5)

### Challenges of social interactions in cafés

Cafés were described as environments in which many possible challenging social interactions might occur; with companions, with other café patrons, or with café staff. Some of these described interactions were more hypothetical in nature or concerned responses to interactions which were internal to the individual with an ED, including fears of difficult comments and perceived judgement from others (see 3.6.1), as well as self-comparisons to other people (see 3.6.2). Others concerned direct interactions with other people in the café including eating with others (see 3.6.3) and having difficult conversations (3.6.4).

#### Fear of difficult comments and perceived judgement from others

Fear of difficult comments and perceived judgement from others was a predominant theme amongst all stakeholder groups, with many recognising this as a significant cause of anxiety and distress (see 3.3). Both fear of comments and perceived judgement related to three key areas of food choices, eating behaviours, and appearance (Table 3):

**Table 3:** Example participant quotes relating to fear of difficult comments and perceived judgement from others

	Fear of difficult comments	Fear of judgement
Food choices	*“…the interactions patients have with cashiers they’ve found quite difficult. Say I’ve had people either offer them free food because they look underweight I’m assuming or make comments like, ‘oh is that all?’ or ‘Gosh, that’s a lot of food.’”* (Cl-4)	*“…it was hard for me to say what I wanted sometimes because I would feel judged and I would think that I would be judged based on what I eat.”* (PWLE-7)
Eating behaviours	*“…a lot of times you’re worried about what other people are going to say or if you’re appearing quite anxious around food, you’re worried about are people going to comment? Are people going to comment on…I don’t know, if you’re taking a long time to start eating or something like that.”* (PWLE-8)	*“…the fear of judgement of people are looking at what you’re eating, the way you eat, how you eat, how long it takes you to eat, all those sorts of things…”* (Cl-5)
Appearance	*“…there’s so many comments and they also quite often make comments on my appearance when I am standing there and they are like, ‘you should have some cake?’”* (PWLE-10)	*“She would become really panicked with too many people around and she’d think that people were looking at her, staring at her because she’s fat even though, obviously, they’re not”* (PC-3)

Regarding difficult comments, participants suggested that even the *anticipation* of potential comments from others in the café was challenging; *“…a lot of times you’re worried about what other people are going to say…”* (PWLE-8), but that challenges arose also from the *distressing emotions that follow* a difficult comment: *“…I have had experiences of people commenting about what I am eating and that has always been the thing that has sort of sent me on a spiral.” (*PWLE-8).

Meanwhile, fear of judgement was described as an internalised worry that others in the café were likely to be judging them: *“…are they going to judge me for what I’m ordering, are they going to judge me for my weight, are they going to judge me for how I look, what are they going to think of me.”* (Cl-2). This intense worry may be related to the participants’ descriptions of a heightened sense of being *“perceived”* (PWLE-2) and worries of *“are they watching me”* (Cl-2).

#### Self-comparisons to other people

Participants described challenging *self-comparison*s with other people (both strangers and companions) regarding appearance and food choices, particularly with those of a similar age: *“…the comparison element of being around other people, body checking, thinking ‘oh the person at the next table is smaller than me’. And looking at what other people are eating, it’s like ‘why have they got a salad and I’ve got a cake’…”* (Cl-2). Multiple participants described a competitive nature to their body size comparisons: *“…if someone else in the café is there, you don’t have to know, like I can see is slimmer than me, there’s an instant, I can feel a sense of jealousy.”* (PWLE-2).

#### Challenges of eating alone and with others

Challenges were identified as arising both from eating alone and with others, with personal preferences appearing to inform which scenario participants preferred: *“Eating alone is a challenging thing in a café but also eating with peers and friends or maybe a romantic partner could also be quite challenging…”* (Cl-1). Eating with others could involve *“…dealing with social expectations…”* about food choices (Cl-6), pressure to eat more, and the difficulty of going for *“a meal with someone who doesn’t order, doesn’t eat or like has something different to kind of what you’re having”* (PWLE-11). This also challenged the secrecy of ED: *“…you don’t want to tell people and they’re not going to get it and there’s lots of shame.”* (PC-2).

However, the majority of participants also identified ways they felt supported by companions which made it easier than eating out alone, through providing *“some like meal time support or post meal support”* (Cl-2), offering encouragement, suggesting food choices or an option to share food, and *“just chit chatting. She found that really helpful and really distracting”* (PC-2). The majority felt most supported by a family member or friend who knew about their ED: *“It’s someone familiar that even with everything else that’s going on and everything that’s different, you can still kind of rely on that friend or family member.”* (PWLE-15). However, some parent/carers and clinicians felt that eating with family posed its own challenges due to heightened emotional involvement: *“…we probably put pressure on her with our anxiety…”* (PC-2), and that early in recovery it could be more beneficial to be supported by a trusted and experienced professional: *“…they could help guide her through it better than we could because we don’t know, we’re not an expert on it.”* (PC-3).

Going alone was described as a barrier to being able to even go to a café: *“I don’t think the confidence will be there to go in on your own”* (PC-3), or resulting in not being able to eat or drink anything: *“I don’t know whether it was motivation or whether things would actually just come up in practice and they wouldn’t be able to do what they’d planned.”* (Cl-6). One PWLE described the added difficulty of eating something that they had chosen themselves instead of being with someone else who could choose for them: *“if someone chooses something for you, and places it in front of you, that’s their fault, and their doing. But if you choose it yourself, then your brain gets more angry at you.”* (PWLE-4).

#### Challenging interactions and specific topics of conversation

Interacting with café staff and patrons was described as an additional challenge which required *“…dealing with the sensory overload of talking while trying to manage anxieties about eating, while trying to appear completely normal.”* (Cl-6). Several participants commented on *“…the way the eating disorder can kind of exacerbate any kind of social anxiety…”* (Cl-2). Participating in or overhearing conversations was particularly difficult if it involved *“scary”* (PWLE-2) topics such as food and nutrition, exercise, or diet culture: *“…we do talk about food casually…”* (Cl-4) and *“…if someone is saying they’re being ‘good’, that’s really tough to deal with…”* (PWLE-8).

### Challenges of the process of choosing and consuming food and drink

For individuals with ED, we might expect that food poses a significant challenge *“because that’s what it is, a fear of eating, and fear of food”* (PC-1). Yet participants provided insights into a series of challenges relating to food and drink beyond their actual consumption, from looking at a menu, decision-making, ordering, and then finally eating and drinking.

#### Looking at menus and calorie counts

Looking at a menu was a common challenge, which was in large part associated with the display of calories, causing anxiety and fear: *“She was too afraid to even look at the menu because the eating disorder voice was telling her, ‘You’re fat enough. There’s too many calories on this’”* (PC-3). Multiple participants recognised that people with ED often know calorie content of food items regardless, but that displaying this information on menus *“…can make people stuck”* (PC-2): *“…you can see her literally, the cogs in her head going round with the calories adding up”* (PC-2). Others highlighted their biased attention towards calorie information so that *“…you’re constantly focusing on that, and everything around you is muted.”* (PWLE-4).

However, many felt that not having calories on menus could be equally challenging: *“…a lot of places do have calorie content listed which I’m in two minds about being good or bad for people… that can cause a lot of anxiety either way.”* (Cl-4). Participants suggested this might depend on the stage of ED recovery and the individual (see 3.4). Several participants recognised the internal conflict of finding both scenarios difficult.

#### Choosing food and drink

Making decisions about what to eat and drink was presented as a complex and overwhelming process, requiring consideration of many factors. Participants described conflict between fear of high calorie foods whilst trying *“…to make the right choice for someone who’s recovering”* (PC-1), choosing *“based on what you want, rather than what your eating disorder wants”* (PWLE-4), and *“picking, say, like, a cake over some fruit”* (PWLE-3). Further conflict arose from worries about choosing something different to companions and a desire for ‘normal’ social eating: *“Is it alright just to have a pile of vegetables and just go for the social event or is that socially unacceptable?”* (PC-4). This complexity was described as *“…it’s like your brain’s being bombarded with all these things in front of you, and you have to make that choice, and your brain is going through all these calculations, whilst you’re also thinking about, ‘Well, what do I want?’… it’s just a constant battle.”* (PWLE-4).

Feeling rushed to make a quick decision by a waiter or other customers was felt to pose a particular challenge by causing *“choice paralysis”* (Cl-4). For this reason, independent cafés for which menus were not available online in advance, or being told a chosen food item was unavailable were particularly difficult scenarios as they involved facing unexpected foods (see 3.2) and rapid decision making: *“It’s like the panic of, ‘Oh, shoot. I need to try and find something else.’”* (PWLE-12).

For many, the complexity of decision-making was exacerbated by a large choice of food: *“the more choice there is, the harder it is…there’s just more options, more things to compare, more things to think about”* (PWLE-6). This was particularly true for those with bingeing behaviours: *“…what happens when too much choice is available and all the options are attractive.”* (Cl-6). However, limited choice also posed a challenge if all options were unappealing or high calorie: *“…big, lardy things like fish and chips can still be a challenge, so she wouldn’t go to somewhere that just served that”* (PC-2). Limited choice formed part of a broader concern that a café might not have an individual’s ‘safe foods’ or those on their meal plan, forcing them to face ‘fear foods’ or ‘triggering foods’ for binge-purge behaviours: *“…they will have a specific thing they’re really intensely worried about…like if full fat milk was going to be used…”* (Cl-4) or *“…not having, like, the diet version of a drink…”* (PWLE-3).

#### Ordering food and drink

The act of ordering food and drink caused particular anxiety for some. Some feared being judged about their order (see 3.6.1), whilst others described *“the social anxiety of having to order something from somebody…”* (Cl-5), and that *“…asking for food and actually saying those words is difficult.”* (Cl-6).

Uncertainty about how to order in a particular café appeared to be a key issue (see 3.2): *“…not like knowing how the café works because some you just sit straight down, others you have to wait to be seated and you order at the table…that can really spike anxiety if we don’t know what we’re doing”* (PWLE-9).

#### Eating and drinking

After navigating the challenges of choosing and ordering, participants described the stage of consuming the food and drink presented to them as a final significant challenge: *“when it turns up like the person doesn’t like it and it just feels like, ‘Oh God, I’ve ordered something that I can’t deal with.’” (PWLE-12).* For many, this required incremental progress towards managing a meal: *“For her, just to even go back into a café and drink a tiny little cup of tea was incredible and then that moved on.”* (PC-3). One clinician recognised that those with binge-eating behaviours might in contrast have: *“…this fear that when they start eating they can’t stop.”* (Cl-1).

Participants highlighted that these fears of eating related to concerns about calories, potential disappointment in the food, and impacts on emotions and food intake later in the day: *“going out for food, as well, can affect the rest of your day, if you’re thinking about what you ate there, and then that might affect, like, what you want to eat later”* (PWLE-3). Difficulty eating in cafés also related to lack of control over portion sizes or quantities of ingredients: *“…if you’ve got an eating disorder, then you’re likely to be very specific about the way that your food is prepared, knowing exactly the amounts you’ve got… The element of control is taken away so it’s going to be absolutely terrifying.”* (Cl-5). This was described as a consequence of the rigidity of ED, and contributes to the overarching theme of the challenge of facing the unexpected and unknown (see 3.2).

## Discussion

ED are characterised by problematic eating or weight-control behaviours, and negative beliefs about weight, shape and eating [4, 5]. We might therefore expect people with ED to experience challenges eating in cafés. Yet our participants outlined a complex series of food- and drink-related challenges beyond actual consumption. These challenges were often driven by a fear of deviating from the rigid cognitive patterns and behavioural routines that underpin chronic restrictive ED [33]. Addressing deficits in cognitive flexibility and decision-making observed in people with ED [34], and creating treatment plans that encourage more spontaneity and flexibility [35] may therefore support people to return to social eating.

A novel finding was the frequency with which participants described the sensory stimuli, contents and layout of the café environment as being a challenge. The challenges of cafés were described as highly interlinked, such that a busy, noisy café environment amplifies the challenges surrounding food and drink, by raising anxiety and impairing the ability to attend to a difficult task. There is some evidence to suggest that people with ED experience heightened sensory sensitivity, including to taste, smell and sound [36, 37]. However, for some individuals, these experiences may reflect comorbid Autism Spectrum Disorder or Attention Deficit Hyperactivity Disorder [37, 38]. A busy environment was also described as interacting with the social challenges of a café. Participants built a picture of heightened awareness of being observed and sensitivity to perceived negative affect, adding to a body of evidence around the social functioning of individuals with ED, including lower positive self-evaluation [39] and consistent fear of judgement with increased sensitivity to perceived criticism or rejection [13]. Several participants described these difficulties as resulting from an interaction between their ED and social anxiety, consistent with evidence of high comorbidity, and that difficulty eating and drinking in public are key symptoms connecting these disorders [10]. This therefore highlights the importance of considering not only the downstream symptoms in potential interventions, but also the underlying cognitive factors and comorbidities.

Underpinning many of the identified challenges was a sense that cafés are unpredictable. Fear of an unknown café environment, unknown foods, and unexpected social interactions may be a manifestation of heightened ‘intolerance of uncertainty’ in ED [33, 40, 41]. For some, disordered eating behaviours may act as a coping mechanism to reduce uncertainty [40, 41]; thus our participants described increased difficulty choosing and consuming food in an uncertain café environment. Our participants also described limited capacity to face these tasks when feeling overwhelmed. Difficulties with emotional regulation, including maladaptive strategies such as avoidance and rumination play an important maintaining role in ED [42], and therefore may be key interventional targets to support people with ED to return to social eating.

### Implications for research and practice

Although this study focused on challenges faced in a café environment, many of these challenges relate more broadly to any social eating scenario such as eating out with friends, peers at school, or family at home. With an improved understanding of some of the specific challenges young people with ED face in these scenarios, and the complex interactions between them, clinicians and parents/carers will be able to provide more targeted support and interventions. Some of the challenges identified, particularly relating to social interactions and fear of judgement from others, provide a broader insight into the challenges that young people with ED face in any public scenario. Given this magnitude of impact on daily life, these findings offer promising targets for intervention development, and will inform the design of a VR graded-exposure café intervention (see study protocol: reference [26]) to support people with ED.

### Strengths and limitations

Our sample included a diversity of genders, ages, and ED amongst PWLE participants. However, despite purposive sampling, individuals with binge-eating behaviours, males, and those of non-white ethnicity were under-represented—a common limitation within ED research [43, 44]. Several participants were able to provide deep insights by drawing on experiences of both supporting people with an ED, and their own lived experiences. It was not possible to always identify which standpoint participants were drawing from for individual quotes, and it was felt attempting to delineate this would not accurately reflect the way each experience will have mutually informed their overall perspectives.

Individuals were able to participate in either focus groups or 1:1 interviews which may have facilitated more open discussion about sensitive topics. This did however reduce the number participating in focus groups; these discussions can be very productive in exploratory research, leading to new ideas and topics [27]. The breadth of perspectives and expertise brought to the analysis by the three researchers was a further strength, incorporating both clinical and research expertise in child and adolescent psychiatry, psychology, and ED, as well as lived experience of ED. In particular, the positionality of LS as both a clinician and someone with lived experience of ED will have shaped the thematic analysis. Medical training will have shaped LS’ understanding of ED as a biopsychosocial phenomenon, whilst lived experience will have enabled a deep empathic and nuanced engagement with participants’ experiences.

## Conclusions

This study sought to explore the perspectives of PWLE, parents/carers and clinicians on the challenges that people with ED experience in café environments, in the context of designing a novel VR café intervention. It highlighted the interacting challenges facing people with ED, many of which relate more broadly to other social eating scenarios and public settings. These findings will enable those supporting someone with an ED to have a deeper understanding of the challenges faced in social eating scenarios and provide more targeted support, and will inform the development of novel interventions.

## Supplementary Information

Additional file1 (DOCX 17 kb)

## Data Availability

The dataset supporting the conclusion of this article is available to bona fide researchers on request via the University of Bristol data repository, data.bris, at 10.5523/bris.2q31p47jp2qsa2q6yywu0kk6op.

## Abbreviations

AN: Anorexia nervosa
ARFID: Avoidant restrictive food intake disorder
BED: Binge eating disorder
BN: Bulimia nervosa
Cl: Clinician
ED: Eating disorder
OCD: Obsessive-Compulsive Disorder
OSFED: Other specified feeding or eating disorder
PC: Parent/carer
PWLE: People with lived experience
SAD: Social anxiety disorder
TA: Thematic analysis
VR: Virtual reality

## Footnotes

1 1.Clinician participant

2 2.Person with lived experience participant

3 3.Parent/carer participant
