# Supplementary material for: The challenges of eating out for young people with eating disorders: a thematic analysis of the perspectives of young people, parents and carers, and clinicians
Source: J Eat Disord. 2025 Dec 16;14:19. doi: 10.1186/s40337-025-01471-z (PMC12821942; doi:10.1186/s40337-025-01471-z)
Supplement: Supplementary file 1 — Additional file1 (DOCX 17 kb) [file 40337_2025_1471_MOESM1_ESM.docx]

Topic Guides for Interviews and Focus Groups

**Topic guide: Focus Groups on VR in Eating Disorders - PWLE**

Ground rules: we are aiming to create a supportive and safe environment. Please consider how what you say might affect others, and in particular don’t mention current or previous weights; please let everyone speak; everything remains in the group; please put your virtual hand up if you want to say something; we might ask individuals if they have anything to add, to give everyone a chance to speak – it’s fine to say no if you don’t. This isn’t a therapy session and we can’t offer individual advice. We will routinely send everyone a debrief sheet with contact details at the end of the session in case taking part raises any issues for you. If you want to leave during the session that’s fine – it would be helpful if you could send us a private message on the chat – we will follow you up with a call to check you’re ok.

***Introductions (5 mins)***

***Background***

What they understand by the term “Virtual Reality”

Any personal experiences of Virtual Reality environments

(Screen-sharing image of VR headset to illustrate this)

***VR Café Environment***

Set scene of idea of a VR café as a way of trying out challenges & reiterate considering how what you say may affect others.

How would you want the café to be represented?

How realistic would you want it to be?

What would make it realistic for you?

***VR Café Challenges***

What might be challenging in a café for someone with an ED?

What might be easiest/harder/most difficult?

What would help make challenges easier? (e.g. things others say or do, less busy, being with someone (who?), ordering “safe” things)

What would make challenges harder (e.g. things others say or do, busier, being with someone, ordering more challenging things)

How would you want to face the challenges? (e.g. Would you want to be able to select them? Or be surprised by a random challenge?)

How would you want to be represented in a café scenario?

How long would you want the experience to last?

How would you like it to end?

***Integration with future treatment (Highlight focus on VR)***

How might VR interface with other treatment for eating disorders?

What might the role of a clinician be?

At what stage of treatment do you think this might be most useful?

Do you have any concerns with VR treatment generally?

Is VR treatment something you would/would have been interested in doing during your recovery/treatment?

What do you think would be the best environment to do the VR in? (e.g. home, clinic, in groups, individually etc.)

Would you want to have the experience on your own, with a friend/family member present, or a clinician?

Would it be a one-off, or something you’d repeat?

Do you think you could take what you learnt in a VR scenario into real life?

***Finishing***: Check in with participants about final comments; thank participants; confirm that debrief sheet will be sent out; check if any concerns; check if would like to receive summary of research findings.

**Topic guide: Focus Groups on VR in Eating Disorders – Parents/Carers**

Ground rules: let everyone speak; please respect each others’ privacy; please put your hand up if you want to say something; we might ask individuals if they have anything to add – this is to give everyone a chance to speak – it’s fine to say no if you don’t have anything to add.

We will routinely send everyone a debrief sheet with useful details at the end of the session.

If you want to leave during the session that’s fine – it would be helpful if you could send us a private message on the chat to let us know – we will call to check you’re ok.

***Introductions***

***VR in General***

What they understand by the term “Virtual Reality”

Any personal experiences of Virtual Reality environments

(Screen-sharing image of VR headset to illustrate what VR is)

***VR Café*** ***Environment***

(Researchers will set the scene of the idea of a VR café as a way of trying out challenging scenarios)

How would you want the café to be represented?

How realistic would you want it to be?

What would make it realistic for you?

***VR Café Challenges***

What might be challenging in a café for someone with an ED?

(ideas if prompts needed: being in the environment, how busy it is, managing anxiety, ordering different items, others’ responses)

What would be useful to practice in a café setting?

What would make the challenges easier?

(e.g. things others say or do, less busy, being with someone (who?), ordering “safe” things)

What would make the challenges even harder?

(e.g. things others say or do, busier, being with someone, ordering more challenging things)

What would make challenges more beneficial?

How should challenges be chosen? (e.g. You can select them? Or be surprised by a random challenge?)

How long should the experience last?

How should it end?

***Integration with future treatment***

Do you think a VR café setting could fit in with other treatment for eating disorders?

What might the role of a clinician be?

At what stage of treatment do you think this might be most useful?

Do you have any concerns with VR treatment generally?

Is VR treatment something you would/would have been interested in your child/adolescent doing during their recovery/treatment?

What do you think would be the best environment to do the VR in? (e.g. home, clinic, in groups, individually etc.)

Would you want to have the experience on your own, with a friend/family member present, or a clinician?

Would it be a one-off, or something to repeat?

***Finishing***: Check in with participants about final comments; thank participants; confirm that debrief sheet will be sent out; check if any concerns; check if would like to receive summary of research findings.

**Topic guide: Focus Groups on VR in Eating Disorders - Clinicians**

Ground rules: let everyone speak; please respect each others’ privacy; please put your hand up if you want to say something; we might ask individuals if they have anything to add – this is to give everyone a chance to speak – it’s fine to say no if you don’t have anything to add.

We will routinely send everyone a debrief sheet with useful details at the end of the session.

If you want to leave during the session that’s fine – it would be helpful if you could send us a private message on the chat to let us know – we will call to check you’re ok.

***Introductions***

***VR in General***

What participants understand by the term “Virtual Reality”

Any personal experiences of Virtual Reality environments

(Screen-sharing image of VR headset to illustrate what VR is)

***VR Café***

(Researchers will set the scene of the idea of a VR café as a way of trying out challenging scenarios)

What might be challenging in a café for someone with an ED?

(ideas if prompts are needed: being in the environment, how busy it is, managing anxiety, ordering different items, other peoples’ responses)

What would be useful to practice in a café setting?

What would make the challenges easier?

(e.g. things others say or do, being with someone, ordering “safe” things)

What would make the challenges even harder?

(e.g. things others say or do, being with someone, ordering more challenging things)

What would make challenges more beneficial?

How should be challenges be decided? (e.g. patient can chose their own? Or be surprised by a random challenge?)

How long should the experience last?

How should it end?

***Integration with future treatment***

Do you think a VR café setting could fit in with other treatment for eating disorders?

What might the role of a clinician be?

At what stage of treatment do you think this might be most useful?

Do you have any concerns with VR treatment generally?

Is VR treatment something you would be interested in doing with the patients you treat?

What do you think would be the best environment to do the VR in? (e.g. home, clinic, in groups, individually etc.)

Would it work best with a friend/family member present, or a clinician?

Would it be a one-off, or something to repeat?

Do you think learning from VR scenarios could be taken into real life?

***Finishing***: Check in with participants about final comments; thank participants; check if any concerns; check if would like to receive summary of research findings.
